# Purification and Identification of Novel Dipeptidyl Peptidase IV Inhibitory Peptides Derived from Bighead Carp (*Hypophthalmichthys nobilis*)

**DOI:** 10.3390/foods13172644

**Published:** 2024-08-23

**Authors:** Hanzhi Zheng, Leyan Zhao, Yushuo Xie, Yuqing Tan

**Affiliations:** College of Food Science and Nutritional Engineering, China Agricultural University, Beijing 100083, China; hz432@cornell.edu (H.Z.); lz397@cornell.edu (L.Z.); xy358@cornell.edu (Y.X.)

**Keywords:** bighead carp skin, collagen peptide, DPP-IV inhibitor

## Abstract

Dipeptidyl peptidase IV (DPP-IV) inhibitors are widely used in treating type 2 diabetes due to their ability to lower blood glucose levels. However, synthetic versions often lead to gastrointestinal side effects. This study explores DPP-IV inhibitory properties in peptides from bighead carp skin. Collagen was prepared, hydrolyzed into collagen peptides, and then fractionated for DPP-IV inhibitory activity examination. The most effective fractions were identified, and their peptide sequences were determined. Molecular docking analysis identified nine peptides with potential inhibitory activity, four of which (VYP, FVA, PPGF, PGLVG) were synthesized and tested in vitro. PPGF exhibited the highest potency with an IC_50_ of 4.63 nM, competitively binding to key DPP-IV sites, including ARG125, VAL711, TYR666, and TYR662. Other peptides showed varying effectiveness, with IC_50_ values of 398.87 nM (VYP), 402.02 nM (FVA), and 110.20 nM (PGLVG). These findings highlight bighead carp skin peptides as potent DPP-IV inhibitors with hypoglycemic potential, suggesting a novel avenue for diabetes management using natural peptides. Moreover, this research underscores the utilization of bighead carp by-products, contributing to environmental sustainability.

## 1. Introduction

Bighead carp (*Hypophthalmichthys nobilis*) is a freshwater fish highly valued for its economic impact, producing 3.187 million tons in 2020 [1]. These fish are primarily sold fresh with minimal processing. Notably, the fish’s head is large and fresh, comprising 36% of its total body mass [2]. Bighead carp is favored by consumers for its richness in docosahexaenoic acid (DHA) and eicosapentaenoic acid (EPA), surpassing some marine fish varieties in these nutrients [3]. However, the meat of bighead carp has a fishy odor, which results in the diminishment of consumer acceptance [4]. The processing of bighead carp also generates significant by-products—including skin, viscera, scales, and bones—amounting to about 60% of the fish’s body mass [5]. The skin alone makes up approximately 3% of the total fish weight and is a valuable source of collagen, gelatin, and active peptides [5]. Additionally, the remaining by-products are also used in the production of fish oil, pet feed, and gluten peptide powder. Without proper management, these by-products are often discarded or landfilled, leading to resource waste and environmental pollution.

Diabetes is a chronic disease marked by high blood sugar levels and disruptions in fat and protein metabolism due to insulin resistance and deficiency. It is categorized into three types: type 1, type 2, and gestational diabetes [6]. As reported by the International Diabetes Federation (IDF), approximately 537 million adults (aged 20–79 years) were living with diabetes in 2021, a number expected to rise to 700 million by 2045 [7]. Type 2 diabetes mellitus (T2DM) constitutes about 90% of these cases. Without treatment, T2DM can lead to severe complications such as coronary issues, retinopathy, and nephropathy [8]. Metformin is one of the primary medications used, yet around 30% of patients suffer from side effects such as nausea [9]. Recent research into T2DM’s pathogenesis has led to new treatment approaches, notably the use of DPP4 inhibitors. These drugs slow the breakdown of glucagon-like peptide-1 (GLP-1) by inhibiting dipeptidyl peptidase IV (DPP-IV), which in turn stimulates the release of glucose-dependent insulin from the pancreas to lower blood sugar [10]. Common DPP-IV inhibitors include sitagliptin, linagliptin, saxagliptin, and alogliptin. However, being chemically synthesized, they often carry risks of side effects like upper respiratory tract infections, headaches, nasopharyngitis, and severe allergic reactions [11]. Moreover, using gliptins with sulfonylureas increases the risk of hypoglycemia [12]. Consequently, there has been a shift towards discovering natural DPP-IV inhibitors, which tend to have fewer side effects and maintain effective blood sugar control. Research has identified a wide array of food-derived protein hydrolysates with DPP-IV inhibitory activity, including those from dairy products (bovine, camel, equine), pig skin, fish skin, and legumes such as soybeans, lupins, and quinoa [13,14]. The most potent natural DPP-IV inhibitor, IPI, was isolated from the culture filtrate of Bacillus cereus BMF673-RF1 and discovered to significantly reduce DPP-IV activity [15]. Notably, peptides GPH (285.3 Da) and GPAG (300.4 Da) from pig skin gelatin hydrolysate inhibit DPP-IV with IC_50_ values of 49.6 and 41.9 μM, respectively, likely due to the inclusion of Pro residues in their structure, particularly at the second N-terminal position, surrounded by Leu, Val, Phe, Ala, and Gly [16]. Milk proteins also yield peptides with DPP-IV inhibitory effects, such as IPM, VP, and IPSK [17]. However, the purification of mammalian proteins faces challenges due to religious restrictions and health risks associated with diseases like foot-and-mouth and mad cow disease [18]. Therefore, fish has emerged as a promising alternative for extracting DPP-IV inhibitors, offering advantages in terms of religious acceptability, safety, and reduced risk of zoonotic diseases.

The aims of this study were to identify the peptides with a potent DPP-IV inhibitory effect and the underlying mechanism. The significance of this study includes (1) offering hypoglycemic agents a sustainable approach to pharmaceutical development, reducing reliance on chemically synthesized DPP-IV inhibitors, which are a medicine for type-2 diabetes, and (2) identifying the competitive inhibition mode and specific binding sites through molecular docking to gain insights into the mechanism of action of these peptides.

## 2. Materials and Methods

### 2.1. Materials

Bighead carp skin was sourced from the Beijing Jianxiangqiao Farmers’ Market. Enzymes used in this study included papain from Angie’s Yeast Co. (Yichang, Hubei, China), pepsin from Sigma-Aldrich (Shanghai, China), and trypsin from Novozymes (Beijing, China). The DPP-IV inhibitor assay kit (KA1311) was obtained from Abnova (Taipei, Taiwan, China). Hydrochloric acid and sodium hydroxide were supplied by the Beijing Chemical Plant (Beijing, China). Additionally, Trypan Blue Dye (0.4%), the hydroxyproline (HYP) content detection kit, and PBS were acquired from Solarbio (Beijing, China).

### 2.2. Bighead Carp Skin Collagen Peptides’ Preparation

#### 2.2.1. Protein Recovery Rate

The protein recovery rate is calculated by comparing the hydroxyproline content in the sample to that in the fish skin, using the methods and reagents from the Hydroxyproline (HYP) Content Assay Kit (Solarbio, Beijing, China). The relationship between the protein recovery rate and hydroxyproline content is expressed by an equation:

(1) $$Protein\;recovery\;rate\;(\%)=\frac{hydroxyproline\;content\;in\;extracted\;collagen\;sample}{hydroxyproline\;content\;in\;skin\;material}\times 100\%$$

#### 2.2.2. Preparation of Collagen

Bighead carp skin was immersed in a 0.1 M sodium hydroxide solution at a solid-to-liquid ratio of 1:8 (*w*/*v*) for 15 h and then rinsed with distilled water. The skin was then mixed with a 0.1 M hydrochloric acid solution at a weight-to-volume ratio of 1:50 (pH 2.4). This mixture was stirred at room temperature in an automatic blender for 48 h. The resulting extract, containing impurities, was transferred to centrifuge tubes and centrifuged at 12,000× *g* for 15 min at 4 °C. The clear supernatant was collected, mixed with a 1 M NaCl solution, and stirred for 3 h to precipitate the collagen. The mixture was then centrifuged again for 10 min at 10,000× *g* at 4 °C. The precipitate was retained for dialysis; the proteins retained in the dialysis membrane were collected and lyophilized.

#### 2.2.3. SDS-PAGE

According to previous research, the collagen sample was dissolved in a 0.02 M sodium phosphate solution (pH 7.2) containing 8 M urea [19]. The sample was then equally mixed with reducing and non-reducing buffers. Electrophoresis was conducted using a separator gel with 7.5% acrylamide and a stacking gel with 5% acrylamide, starting at an initial voltage of 150 V. After electrophoresis, the gel was stained and then destained on a shaker.

### 2.3. Hydrolysis of Collagen and Measurement of Molecular Weight Distribution

#### 2.3.1. Hydrolysis of Collagen

The selection of hydrolase considered the digestive system of the human gastrointestinal tract. To ensure the potential stability of hydrolyzed products during digestion, pepsin and trypsin were selected to avoid the inactivation of peptides by further enzymatic digestion. Additionally, proteolytic digestion in vitro treated with papain showed a significantly higher DPP-IV inhibition rate compared to that treated with neutral protease, trypsin, or pepsin [20]. Consequently, papain was also chosen as one of the complex enzymes due to its superior performance.

Collagen was mixed with deionized water at a ratio of 1:20 (*w*/*v*), with the pH adjusted using HCl and NaOH. This mixture was placed in a temperature-controlled water bath. There were two ways of adding complex enzymes. One was papain (pH 7.0, 50 °C), pepsin (pH 2.0, 37 °C), and trypsin (pH 8.0, 37 °C). The other was pepsin (pH 2.0, 37 °C) and trypsin (pH 8.0, 37 °C). These two combinations were performed separately. The enzyme-to-sample mass ratio (enzyme/sample mass) was 0.4% for pepsin, 2.6% for papain, and 4% for trypsin. During the hydrolysis process, each type of enzyme was added sequentially as the temperature and pH conditions reached optimal levels, with a one-hour interval between them. Afterward, the mixture was placed in a 95 °C water bath for 15 min to deactivate the enzymes. Hydrolysates were then lyophilized into powder using a freeze dryer (FD-1PF, Beijing Detianyou Co., Ltd., Beijing, China).

#### 2.3.2. Measurement of the Molecular Weight Distribution

The molecular weight distribution of the hydrolysates was determined using high-performance liquid chromatography (HPLC) [21]. The hydrolysates were dissolved in a solution of 45% acetonitrile with 0.1% (*v*/*v*) trifluoroacetic acid (TFA) to prepare a sample solution with a concentration of 2 mg/mL. This solution was then filtered and injected into a TSK G2000 SWXL gel exclusion chromatographic column (7.8 × 300 mm, TOSOH, Tokyo, Japan). The mobile phase was isocratically eluted at a flow rate of 0.5 mL/min. Absorbance values of the samples were detected at a wavelength of 220 nm using a UV detector. The molecular weight distribution of the hydrolysates was determined by correlating the absorbance values with a standard curve.

### 2.4. Isolation and Purification of Collagen Peptides

#### 2.4.1. Ultrafiltration of Hydrolysates

Hydrolysates of bighead carp skin collagen were separated by ultrafiltration using an Amicon ultrafiltration cup (UFSC20001, Millipore, Billerica, MA, USA). The solution, prepared with deionized water at a concentration of 25 mg/mL, was passed through a 3 kDa regenerated cellulose ultrafiltration membrane to isolate fractions with molecular weights of less than 3 kDa. These fractions were collected and subsequently lyophilized.

#### 2.4.2. RP-HPLC Purification

Fractions with molecular weights below 3 kDa were dissolved in ultrapure water to prepare a solution with a concentration of 100 mg/mL. Subsequently, they were purified by RP-HPLC (Shimadzu Technologies Co., Ltd., Kyoto, Japan) using a Kromasil C18 semi-preparative column (4.6 × 250 mm). The mobile phase consisted of ultrapure water containing 0.1% trifluoroacetic acid (mobile phase A) and acetonitrile containing 0.1% trifluoroacetic acid (mobile phase B). The gradient elution conditions were as follows: from 0 to 10 min, phase B was maintained at 10%; from 10 to 15 min, phase B was increased from 10% to 15%; from 15 to 20 min, phase B was increased from 15% to 20%; from 20 to 25 min, phase B was increased from 20% to 30%; from 25 to 30 min, phase B was increased from 30% to 50%; from 30 to 35 min, phase B was increased from 50% to 65%; and from 35 to 40 min, phase B was reduced back to 5%. Each injection volume was 100 μL, and the elution rate was set at 1.5 mL/min. Based on the multiple peaks separated by RP-HPLC, the collagen peptides were divided into different fractions. The DPP-IV inhibitory activity of these fractions was determined as described in Section 2.5.

### 2.5. Determination of DPP-IV Inhibitory Activity

The DPP-IV inhibition rate and the corresponding IC_50_ of each component were determined using the DPP-IV inhibitor screening assay kit (KA1311, Abnova, Taipei, Taiwan, China). For each component, extracts were mixed with buffer solution at various concentrations (1 mg/mL, 0.1 mg/mL, 0.05 mg/mL, 0.01 mg/mL), DPP-IV, and the substrate (Gly-Pro-AMC solution) in 96-well plates. The reaction was conducted at the normal human body temperature of 37 °C for 30 min. Free AMC (7-amino-4-methylcoumarin) was quantified using a multifunctional fluorescent zymography assay, with excitation light at 350 nm and emission light at 460 nm, and expressed as fluorescence intensity. The positive control utilized was 1 μg/mL of selegiline. A standard curve for all inhibition rates was plotted, and the maximum half inhibitory concentration (IC_50_) value of each peptide was determined using GraphPad Prism 9.5.1.

DPP-IV inhibition (%) = {(F initial activity − F sample)/F initial activity} × 100%(2)

### 2.6. Identification of Peptide Sequences by Nano-HPLC-MS/MS

The fractions exhibiting high DPP-IV inhibition were selected for further analysis, and the peptide sequences were characterized using an Orbitrap Q-Exactive Plus mass spectrometer (Thermo Fisher Scientific, Waltham, MA, USA) coupled with a high-resolution tandem EASY-nanoLC 1200. The elution column utilized was an Acclaim PepMap C18 (75 μm × 25 cm), with mobile phases consisting of ultrapure water containing 0.1% formic acid (mobile phase A) and 80% acetonitrile containing 0.1% formic acid (mobile phase B). The elution program involved a linear gradient of mobile phase B increasing from 2% to 35% over 47 min, with an elution rate of 300 nL/min. The Orbitrap Q-Exactive Plus mass spectrometer automatically switched between MS and MS/MS acquisitions. The MS scanning range was set at 200–2000 *m*/*z* with an MS/MS scanning resolution of 70,000. The HCD-MS/MS scanning resolution was set at 17,500, and the electrospray voltage was maintained at 2 kV. The mass spectrometry data were analyzed by searching the Uniprot-Hypophthalmichthys nobilis (version 2023, 431 entries) database for target amino acid sequences. Unitiprot was used to search four databases: the database A0A077B3P8 for the protein α_1_-type I collagen of silver carp; the database A0A2H4ZEX8 for the protein α_2_-type I collagen of silver carp; the database A0A8C2FYG1 for the protein α_2_-type I collagen of common carp; and the database A0A9J7YD14 for the protein α_1b_-type I collagen. The hydrophobicity of the identified peptides was obtained from the Pepdraw online tool (http://www2.tulane.edu/%7ebiochem/WW/PepDraw/, accessed on 25 December 2023).

### 2.7. Molecular Docking

Human dipeptidyl peptidase IV (Protein Data Bank ID 1J2E), an enzyme involved in the degradation of both gastric inhibitory polypeptide (GLP-1) and glucagon-like peptide (GIP), was selected as the protein binding site for the studied peptides derived from bighead carp [22,23]. The spatial structure of 1J2E was obtained from the Protein Data Bank (PDB) (https://www.rcsb.org/structure/1j2e, accessed on 21 November 2023) [24]. Ligand peptides were simulated using Discovery Studio 2021 (BIOVIA). Initially, the DPP-IV protein structure was prepared by deleting water molecules and retaining one of the monomers for pre-docking preprocessing, as recommended by Systèmes [25]. Subsequently, the 3D structural adjustment of the peptide molecules was conducted using the Avogadro computer tool [26]. For blind molecular docking, HPEPDOCK 2.0 software was employed [27]. Following the docking process, Discovery Studio was utilized to create a 2D schematic illustrating the binding mode of the peptide and DPP-IV molecules. This schematic aids in visualizing the interactions between the ligand peptides and the receptor DPP-IV, providing insights into their binding modes and potential inhibitory mechanisms. The lPl peptide segment emerged as the natural peptide with the most potent inhibitory potential against DPP-IV [13]; thus, it was selected as a positive control for comparison with the peptides chosen in this study.

### 2.8. Synthesis of Peptides

According to the criteria, synthetic peptides were screened to further analyze DPP-IV inhibitory activity in vitro, and the criteria were as follows: (1) less than 5 amino acid residues; (2) high abundance in area 4 or 5; and (3) presence of hydrophobic amino acids at C-terminal or N-terminal. Synthetic peptides were obtained from Shanghai Royobiotech Co., Ltd. (Shanghai, China), with a purity of over 98%.

### 2.9. Determination of DPP-IV Inhibition Pattern

The pattern of DPP4 inhibition of synthetic peptides was further analyzed using Lineweaver–Burk plots. Images were plotted at different concentrations of substrates (20, 15, 10, 5, 1 μM) and peptide solution (concentration was greater than IC_50_, less than IC_50_, and 0) with 1/[S] as the X-value and 1/V as the Y-value. The mode of inhibition of DPP-IV by the synthetic peptide was determined based on the intercept of the Lineweaver–Burk curve.

### 2.10. Prediction of Toxicity and Digestibility

The potential toxicity and allergenicity of the synthesized peptides were predicted using ToxinPred (https://webs.iiitd.edu.in/raghava/toxinpred/, accessed on 27 February 2024) and AllerTOP v.2.0 (https://www.ddg-pharmfac.net/AllerTOP/, accessed on 29 February 2024), respectively.

In addition, an in vitro analysis of the digestive stability of synthesized peptides in the gastrointestinal tract was conducted by using PeptideCutter (http://web.expasy.org/peptide_cutter/, accessed on 29 February 2024), selecting the enzymatic cleavage sites of chymotrypsin—low specificity (C-term to [FYWML], not before Pro); pepsin; and trypsin for prediction.

### 2.11. Statistical Analysis

The DPP-IV inhibitory activity assay was performed in three parallel groups, and IC_50_ data were expressed as the mean ± standard deviation (S.D.). Data were analyzed using SPSS 20.0 software (SPSS Inc., Chicago, IL, USA) for unifactorial and bifactorial analyses, and the significance of the data was indicated by *p* < 0.05.

## 3. Results and Discussion

### 3.1. SDS-PAGE, Recovery Rate, and Molecular Weight Distribution of Prepared Bighead Carp Skin Collagen

The recovery rate of bighead carp skin was calculated to be 48%, which is higher than other recovery rates from the collagen of other fish, like Nile perch skin [28], bigeye snapper skin [29], and hydrochloric-acid-extracted catfish skin [19]. But it does not exclude the possibility that the extracted collagen contains impurities. The SDS-PAGE of prepared collagen is shown in Figure S1, indicating four chains corresponding to the γ-chain (386.1 KDa), β-chain (258.2 KDa), α1-chain (165.9 KDa), and α2-chain (147 KDa). This pattern of bands is consistent with collagen bands observed in channel catfish collagen [19]. However, it is noted that in some studies, such as the analysis of bighead carp skin collagen, three α bands (α1, α2, and α3) were reported [30]. This discrepancy may arise from the fusion of the α3 band below the α1 band, making it difficult to distinguish them [31]. In the SDS-PAGE analysis, lane 2 represents a sample treated with reducing super sampling buffer (DTT). Interestingly, despite the addition of DTT, the shape of the bands remained largely unchanged compared to the untreated sample. This observation suggests that the structure of collagen is not significantly affected by DTT treatment, which supports the previous findings [32].

**Figure 1:**
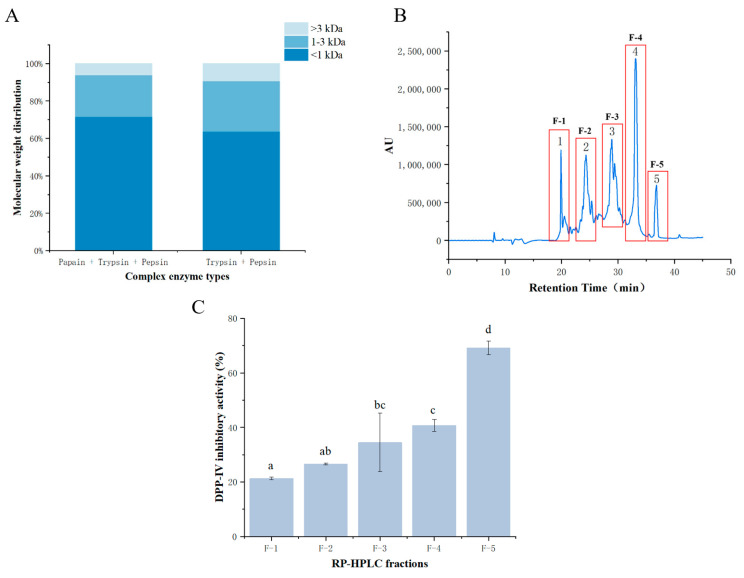
(**A**) Molecular weight distribution of collagen peptides from the skin of bighead carp digested by two kinds of multienzyme. (**B**) Reversed-phase high-performance liquid chromatography of collagen peptides from bighead carp skin < 3 kDa. (**C**) DPP IV inhibitory activity of various fractions of bighead carp skin collagen peptides at different concentrations. Note: The combination of group one contains papain, trypsin, and pepsin; the combination of group two contains trypsin and pepsin. The same lowercase letter (a–d) for different fractions of the collagen peptides at the same concentration indicates no significant difference in inhibitory activity (*p* > 0.05), and the values are expressed as the mean ± standard deviation (*n* = 3).

The findings presented in Figure 1A demonstrate that the majority of the collagen peptides derived from bighead carp skin had molecular weights less than 3 kDa following hydrolysis, particularly in the first combination of complex enzymes, including papain, trypsin, and pepsin. This group of hydrolysates exhibited a prevalence of small-molecular-weight peptides, with 70.07% of the protein peptides being less than 1 kDa and an impressive 93.59% of the protein peptides being less than 3 kDa. This distribution of peptide sizes is significant, as studies have indicated a correlation between molecular weight and protein activity. Specifically, previous research suggests that smaller-molecular-weight peptides often exhibit greater protein activity [33]. Moreover, there is evidence supporting the notion that peptides with molecular weights less than 3 kDa tend to possess higher DPP-IV inhibitory activity compared to peptides with larger molecular weights [34]. Based on these insights, the fractions from group one containing a higher proportion of small-molecular-weight peptides were selected for subsequent experiments. This decision aims to isolate peptide sequences with enhanced DPP-IV inhibitory activity, leveraging the observed correlation between molecular weight and biological activity.

### 3.2. DPP-IV Inhibitory Activity of Fractions after Purification

#### 3.2.1. RP-HPLC of DPP-IV Inhibitory Peptides

The separation of fractions (<3 kDa) obtained from the ultrafiltration of the hydrolysates involved the use of a Kromasil C18 semi-preparative column, employing reversed-phase high-performance liquid chromatography (RP-HPLC) based on polarity. In Figure 1B, the RP-HPLC chromatogram reveals five distinct peaks, corresponding to five components. These components were separated based on their hydrophobicity, with more polar components eluting first, and thus, the components are ranked according to their hydrophobicity, from strongest to weakest: F-5 > F-4 > F-3 > F-2 > F-1. The hydrophobicity of peptides, along with factors such as specific amino acid groups and their positions, plays a crucial role in determining their DPP-IV inhibitory activity, as highlighted in various studies [13]. Therefore, it is hypothesized that fractions with longer retention times on the column, indicating greater hydrophobicity, may exhibit stronger DPP-IV inhibition potential. Specifically, fractions F-3, F-4, and F-5 are anticipated to demonstrate enhanced DPP-IV inhibitory activity based on their longer retention times. To validate the relationship between hydrophobicity and DPP-IV inhibitory activity, the five fractions were collected separately, and their DPP-IV inhibitory rates were determined.

#### 3.2.2. DPP-IV Inhibitory Activity of Fractions

The purification process involving ultrafiltration and RP-HPLC successfully separated bighead carp skin collagen peptides into shorter peptides with smaller molecular weights. As illustrated by the five peaks in Figure 1B, the separated fractions exhibited increasing inhibition rates from F-1 to F-5 at a concentration of 1 mg/mL, reflecting the rising hydrophobicity of the samples. Notably, F-5 demonstrated the highest inhibition rate of 69.24%, indicating its robust ability to inhibit DPP-IV. This finding aligns with the observations of previous research, indicating that peptides with potent DPP-IV inhibition typically contain hydrophobic amino acids [13]. Although the inhibition rates of the other fractions were lower than that of F-5, the differences were not statistically significant. Hence, the determination of the semi-inhibitory concentration (IC_50_) for DPP-IV was essential for further analysis. By calculating the IC_50_ values based on the inhibition rates (as presented in Figure 1C), F-5 exhibited the lowest semi-inhibitory concentration of DPP-IV with a value of 0.54 ± 0.65 (mg/mL), indicating its superior inhibitory activity. F-4 followed closely in terms of inhibitory activity. The IC_50_ values of the five fractions revealed a pattern where the DPP-IV inhibitory activity corresponded to the hydrophobicity of the fractions, ranging from strong to weak (F-5 > F-4 > F-3 > F-2 > F-1), consistent with the earlier speculation. Therefore, the separation of F-4 and F-5 with RP-HPLC resulted in fractions with notably stronger inhibitory activity against DPP-IV, underscoring their potential for therapeutic application in managing metabolic disorders like diabetes.

### 3.3. Identification and Analysis of Peptide Sequences

#### 3.3.1. Nano-HPLC-MS/MS Spectra of Collagen Peptides

**Figure 2:**
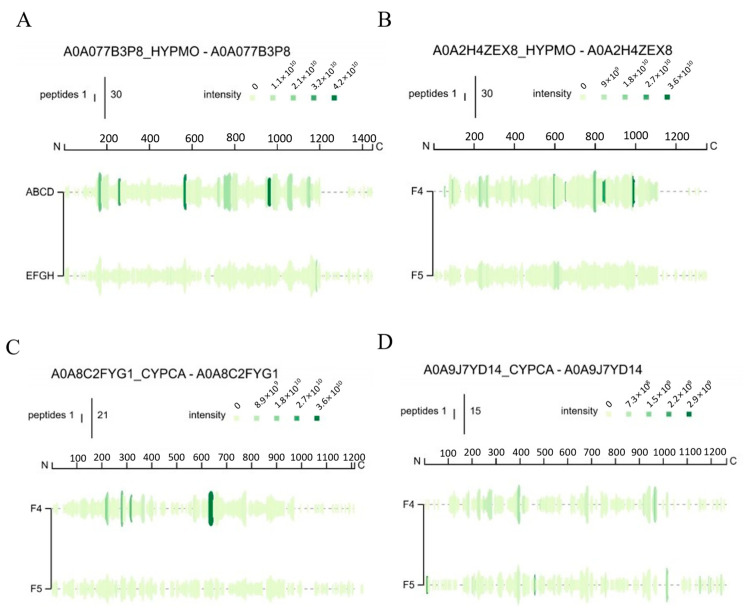
Peptide distribution of collagen peptides from bighead carp (*Hypophthalmichthys nobilis*) skin: (**A**) A0A077B3P8, (**B**) A0A2H4ZEX8, (**C**) A0A8C2FYG1, and (**D**) A0A9J7YD14.

The Peptigrams of F-4 and F-5 are indicated as F4 and F5 in Figure 2, showing that the peptide distributions in F-4 are more complex, which means it has much more peptides with high abundance. The diagram shows that the amino acid sequence of F-4 was widely distributed in Figure 2A, with a coverage of 83%. The peptide fragments were mainly distributed in G150-R205, G749-K811, and G944-R1011. Q561-K571 was mainly QPGVMGFPGPK. In Figure 2B, the peptide fragments are mainly distributed in the latter part of whole protein (G768-G984). The amino acid sequence of A985-P994 is ARGAPGPAGP. According to Figure 2C, peptides are mainly located in the middle of the α2-type I collagen of common carp (G628-R669), with an additional portion distributed in G214-K225 (GLAGDPGPVGVK). However, there is no significant difference in the distribution region in α1b-type I collagen from common carp (Figure 2D), where the amino acid sequence of A388-K398 is APGVMGFPGPK and that of P934-R944 is PAGPSGPAGPR.

Accordingly, N-terminal hydrophobic amino acids, such as His, Tyr, Val, Ile, and Pro, can enhance the specificity of DPP-IV substrates and promote their interaction with DPP-IV, thereby boosting inhibitory properties [35]. Additionally, peptides containing a proline (Pro) residue at the second C-terminal or second N-terminal position are believed to exhibit potential DPP-IV inhibitory activity [36]. Typically, DPP-IV inhibitors consist of fewer than 10 amino acid fragments, though some studies have reported inhibitors with 11 amino acid fragments [37,38]. Notably, potent DPP-IV inhibitory peptides typically range from three to six amino acid residues in length [13,39,40]. Hence, the peptide fragments identified in the database hold significant potential for strong DPP-IV inhibitory activity and can effectively bind to DPP-IV sites, suggesting promising outcomes for docking studies [41].

#### 3.3.2. Molecular Docking of Collagen Peptides

**Figure 3:**
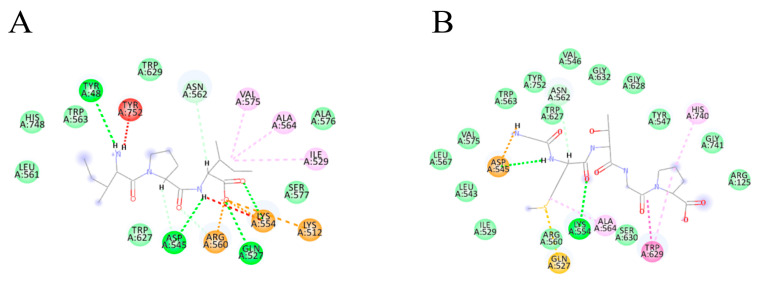

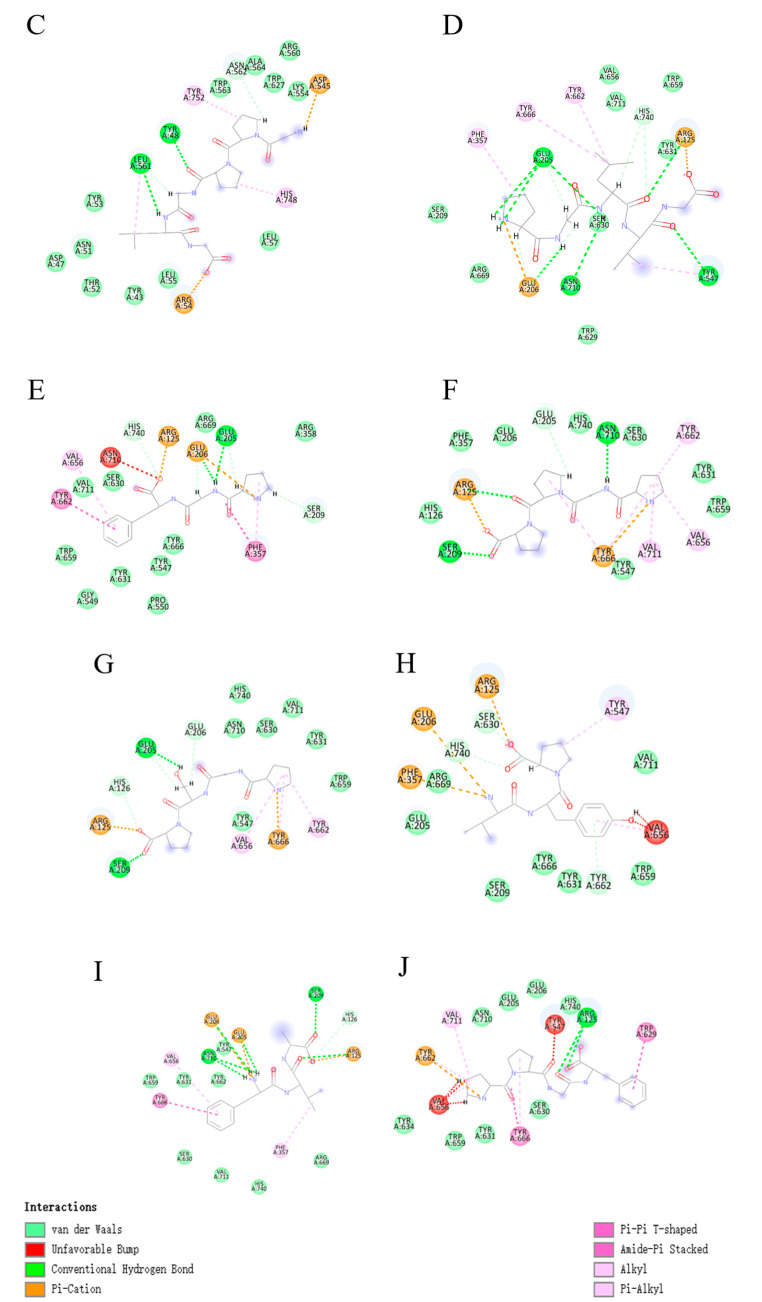
Binding modes of peptides to DPP-IV receptor sites: (**A**) IPI, (**B**) GMTGP, (**C**) GPPGLG, (**D**) PGLVG, (**E**) PGF, (**F**) PGPP, (**G**) PGSP, (**H**) VYP, (**I**) FVA, and (**J**) PPGF; K is a color-coded annotation of the various binding modes.

Figure 3 illustrates the interaction patterns of IPI (control), GMTGP, GPPGLG, PGLVG, PGF, PGPP, PGSP, VYP, FVA, and PPGF with DPP-IV in 2D diagrams. Table 1 provides a summary of hydrogen bonds and pi-alkyl interactions between specific peptides and DPP-IV, along with each peptide’s binding score and hydrophobicity. The results indicate that all selected peptides have lower scores than IPI, the positive control, suggesting their potential inhibitory ability against DPP-IV. Additionally, the results reveal a clear relationship between the docking score and peptide characteristics, including interactions with DPP-IV and hydrophobicity. Regarding interactions, the findings suggest a positive relationship between the hydrogen bond or pi-alkyl interaction and docking score. In other words, peptides engaging in more interactions with DPP-IV are more likely to demonstrate inhibitory ability. This aligns with previous research, which found that peptides forming more hydrogen bonds tend to have lower binding scores [42]. Specifically, FVA and PGLVG demonstrate evidence of the correlation between more hydrogen bonds and better binding. For example, PGLVG forms seven hydrogen bonds with GLU205, GLU206, ASN710, TYR547, and ARG125, while FVA forms six bonds with ASN710, GLU206, GLU205, SER209, and ARG125. PGLVG, forming the highest number of hydrogen bonds with DPP-IV, has the third-lowest docking score among the selected peptides, indicating a close relationship between interactions and binding affinity. The analysis of the table reveals that binding sites such as Glu 205, Tyr 666, and Glu 206 frequently form hydrogen bonds with peptides. However, despite forming only two hydrogen bonds, PPGF exhibits the same lower docking score as FVA. This suggests that, in addition to hydrogen bonds, pi bonds may play a critical role in the binding mechanism for PPGF (Figure 3J). Tyr 666 exhibits a high tendency to form pi bonds with inhibitory peptides. Additionally, peptides with shorter chains seem to demonstrate better binding situations, suggesting an area worthy of further research. In terms of binding positions, DPP-IV consists of two pockets: the S1 pocket (Tyr631, Val656, Trp659, Tyr662, Tyr666, Val711) and the S2 pocket (Arg125, Glu205, Glu206, Phe357, Ser209, Arg358) [41]. Among all peptides derived from fish skin collagen, FVA displays the lowest binding energy with the DPP-IV protein, indicating potent inhibitory efficacy. It is noteworthy that GLU206, GLU205, SER209, and ARG125 collectively constitute the positions forming the S2 pockets, indicating that these residues from the S2 pockets have a strong tendency to form hydrogen bonds with specific peptides.

The primary sequences of peptides were obtained from the results of nano-HPLC-MS/MS. Furthermore, based on information from other research focusing on the characteristics of DPP-IV inhibitory peptides, certain conditions were established to predict which peptides may exhibit inhibitory ability: (1) the presence of Leu-Leu or Pro-Gly at the peptide’s N-terminus [38]; (2) the presence of a Pro residue at the C-terminus [43]; (3) the proximity of hydrophobic residues to the peptide ends [13,44]; and (4) peptides containing Pro, His, or Ala residues in their sequences [45,46].

The observation regarding the exceptional binding situation of FVA, despite its specific amino acid composition, is indeed intriguing. FVA stands out due to the presence of an alanine residue along with hydrophobic amino acids at both the N-terminal and the second N-terminal/C-terminal positions. This unique arrangement might contribute to its distinctive binding characteristics with DPP-IV. Moreover, the finding that the peptide GPPGLG, despite demonstrating the highest hydrophobicity among the selected peptides, did not yield a lower docking score is quite significant. It suggests that while hydrophobicity plays a crucial role in the peptide–DPP-IV interaction, it is not the sole determinant of binding affinity. Other factors such as the specific spatial arrangement of amino acids, electrostatic interactions, hydrogen bonding, and steric effects could also significantly influence the binding affinity between peptides and DPP-IV.

### 3.4. DPP-IV Inhibitory Activity of Synthetic Peptides

The results of mass spectrometry identification guided the screening of peptide sequences based on certain criteria. Four peptide sequences demonstrating superior DPP-IV inhibitory activity were selected for synthesis, and their respective DPP-IV inhibition rates and IC_50_ values were determined (refer to Table 2). Notably, PPGF exhibited the lowest IC_50_ value (4.63 nM), followed by PGLVG (IC_50_ value: 110.20 nM). These findings suggest that these two peptides possess potent DPP-IV inhibitory abilities and are novel discoveries. In the current landscape of research aiming to identify DPP-IV inhibitors in nature, numerous studies are ongoing. However, the IC_50_ values of the four peptides uncovered in this study were smaller than those of previously reported peptides such as YYGYTGAFR (1102.8 μM) from Atlantic salmon [47], YPFPGP (749.2 μM) extracted from milk [35], and WGDEHIPGSPYH (350 μM) from silver carp [48]. This comparison underscores the significant DPP-IV inhibitory activity exhibited by these novel peptides.

The results of PeptideCutter indicated that all synthesized peptide fragments possessed enzymatic cleavage sites, suggesting susceptibility to inactivation via gastrointestinal digestion. However, the presence of fewer enzymatic cleavage sites also implies the potential for functional activity in vivo. Nevertheless, further research is warranted to confirm the potential allergenicity, toxicity, and digestive stability of the synthesized peptides in vivo. In vivo studies are essential to comprehensively evaluate the physiological effects and safety profile of these peptides, ensuring their suitability for therapeutic or functional applications. Additionally, assessing their stability and behavior in a biological context will provide valuable insights into their potential as therapeutic agents or dietary supplements.

The assessment of potential toxicity and allergenicity is crucial in the synthesis of peptides intended for human consumption due to their possible adverse effects on health. Analysis revealed that all peptide sequences, except for VYP, were deemed non-toxic (Table 2). However, VYP was flagged as potentially allergenic. Further allergen testing revealed structural similarities between VYP and a protein with NCBI gene sequence 83754241, a known allergen. The presence of an allergenic potential in VYP could be attributed to the retention of certain structural sequences from the natural protein source during peptide synthesis. This phenomenon is not uncommon, as peptides derived from natural proteins may still contain epitopes capable of triggering allergic reactions. Interestingly, studies have suggested that peptides with small molecular weights have a lower likelihood of containing intact antigenic epitopes [49]. Additionally, it is noted that pentapeptides represent the minimum sequence length for immunorecognition. These findings may indicate that the risk of allergenicity decreases with decreasing peptide size [50].

### 3.5. DPP-IV Inhibitory Pattern

**Figure 4:**
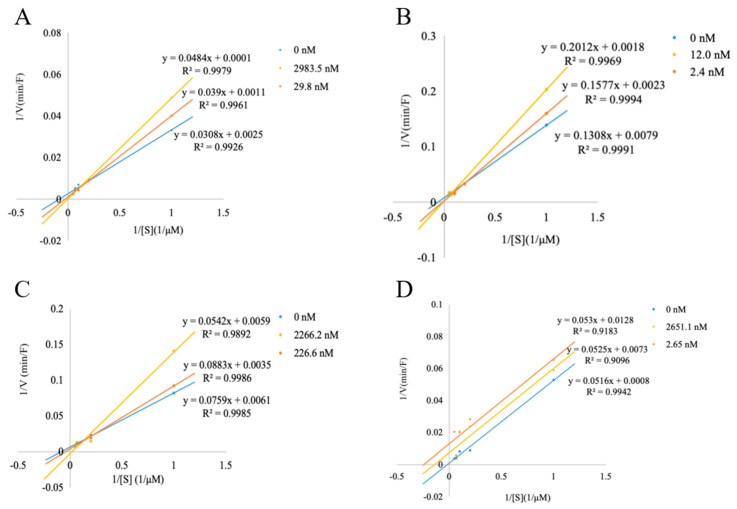
The inhibition kinetics of four synthetic peptides: FVA (**A**), PPGF (**B**), PGLVG (**C**), and VYP (**D**).

The inhibition kinetics of each peptide were explored using Lineweaver–Burk plots, and further analysis was conducted to investigate the DPP-IV inhibitory mechanism of the four synthesized peptides (Figure 4A–D). The concentrations of the peptides varied across greater than IC_50_, less than IC_50_, and 0. Starting with Figure 4A, it is evident that FVA exhibited almost the same Vmax value and increased Km at different concentrations, as reflected by the common intersection point on the *y*-axis, which indicates that the mode of inhibition for FVA is competitive inhibition [51]. Similarly, the Lineweaver–Burk plots for PPGF (Figure 4B) also displayed the same mode of inhibition as FVA, indicating competitive inhibition. But PGLVG (Figure 4C) showed a mixed competitive inhibition mode, in which case the Vmax is reduced because PGLVG can bind to allosteric sites non-competitively, while Km is increased due to the active site reactions of PGLVG and DPP-IV, competing with substrates.

It was suggested that collagen peptides act as competitive inhibitors of DPP-IV, a characteristic attributed to their substrate analogue properties [52]. Moreover, competitive inhibition has been associated with inducing a substrate-like behavior in peptides containing Pro residues at the P2 position, as observed in casein-derived peptides [53]. However, the inhibition pattern observed for VYP (Figure 4D) was notably different, demonstrating uncompetitive inhibition. This divergence in the inhibition mechanism suggests that VYP only binds to enzyme–substrate complexes, compared to the other peptides.

In this study, the PPGF peptide had the lowest IC_50_ value of 4.63 nM, and it was a competitive-inhibition-mode peptide, indicating the strongest inhibition of DPP-IV, while VYP had a relatively weak inhibition of DPP-IV in the uncompetitive inhibition mode. Therefore, it is possible that peptides with different modes of inhibition will also differ in their ability to inhibit DPP-IV.

## 4. Conclusions

This investigation into DPP-IV inhibition revealed that among the nine peptides containing Pro, Ala, and Gly, four exhibited notable DPP-IV inhibitory activity, with FVA, PPGF, PGLVG, and VYP demonstrating particularly strong inhibition. Molecular docking studies provided insights into the binding sites and docking scores, highlighting PPGF and PGLVG as the most potent inhibitors of DPP-IV. The robust DPP-IV inhibitory activity observed in PPGF and PGLVG suggests their potential as natural and novel inhibitors for the treatment of type 2 diabetes. These peptides hold promise as lead compounds for the development of therapeutic agents targeting DPP-IV.

## Figures and Tables

**Table 1:** Docking scores, interaction forces, and binding sites of each peptide with DPP-IV.

Peptide	Docking Score	Hydrophobicity (kcal/mol)	Binding Atoms	Distance (Å)	Interaction
IPI (control)	−105.877	+5.80	TYR48 ASP545 GLN527 LYS544	2.75 2.31 2.23 2.50	Hydrogen bond
			N/A	N/A	π alkyl bonds
GMTGP	−146.637	+9.92	ASP545 LYS544	2.93 2.10	Hydrogen bond
			TRP629 TRP629 HIS740	4.60 5.26 5.27	π alkyl bonds
GPPGLG	−131.363	+10.38	LEU561 TYR48	2.83 2.08	Hydrogen bond
			LEU561 HIS748 TYR752	4.63 4.62 5.27	π alkyl bonds
PGLVG	−153.308	+8.63	GLU205 GLU205 GLU205 GLU206 ASN710 TYR547 ARG125	2.88 2.90 3.07 1.98 2.20 2.73 2.19	Hydrogen bond
			PHE357 TYR666 TYR662 TYR547	4.07 5.18 4.90 4.01	π alkyl bonds
PGF	−149.957	+7.48	GLU206 GLU205	1.66 2.56	Hydrogen bond
			VAL656 PHE357	5.22 4.68	π alkyl bonds
PGPP	−138.128	+9.47	SER209 ARG125 ASN710	1.90 2.76 2.75	Hydrogen bond
			TYR666 TYR666 VAL711 VAL656 TYR662	4.91 5.37 5.23 4.34 4.26	π alkyl bonds
PGSP	−137.463	+9.79	SER209 GLU205	1.28 2.05	Hydrogen bond
			VAL656 TYR666 TYR662	4.88 4.91 4.38	π alkyl bonds
VYP	−143.971	+6.87	TYR662 HIS740 SER630	6.77 4.72 4.25	Hydrogen bond
			VAL656 TYR547	7.25 6.10	π alkyl bonds
FVA	−154.863	+6.23	ASN710 ASN710 GLU206 GLU205 SER209 ARG125	4.79 5.27 5.21 4.82 3.99 4.84	Hydrogen bond
			TYR666 PHE357 VAL656	5.21 5.89 6.96	π alkyl bonds
PPGF	−154.322	+7.62	ARG125 ARG125	5.75 5.91	Hydrogen bond
			VAL711 TYR666 TYR662	6.24 5.88 5.58	π alkyl bonds

Note: the molecular ID of DPP-IV is 1J2E.

**Table 2:** DPP-IV inhibitory activity and biological activity of synthetic peptides.

Peptide Sequences	Allergen FP	ToxinPred	Peptide Cutter	Molecular Weight (Da)	IC_50_ (nM)
VYP	Probable allergen	Non-toxin	2	377.19	398.87 ± 47.43 a
FVA	Probable non-allergen	Non-toxin	1	335.18	402.02 ± 5.28 a
PPGF	Probable non-allergen	Non-toxin	4	416.21	4.63 ± 1.56 b
PGLVG	Probable non-allergen	Non-toxin	3	441.26	110.20 ± 43.06 c

Note: 1, 2, 3, and 4 represent the positions of cleavage sites, and cleavages occur from the right side (C-terminal direction) of the marked amino acid. The same lowercase letter (a–c) for different sequences of the synthetic peptides indicates no significant difference in IC50 (*p* > 0.05), and the values are expressed as the mean ± standard deviation (*n* = 3).

## Data Availability

The original contributions presented in the study are included in the article/supplementary material, further inquiries can be directed to the corresponding author.

## Supplementary Materials

The following supporting information can be downloaded at: https://www.mdpi.com/article/10.3390/foods13172644/s1, Figure S1: SDS-PAGE of collagen.
