# Supplementary material for: Purification and Identification of Novel Dipeptidyl Peptidase IV Inhibitory Peptides Derived from Bighead Carp (Hypophthalmichthys nobilis)
Source: Foods. 2024 Aug 23;13(17):2644. doi: 10.3390/foods13172644 (PMC11394167; doi:10.3390/foods13172644)
Supplement: Supplementary file 1 [file foods-13-02644-s001.zip › foods-3111716-supplementary.pdf]

Supplementary Materials

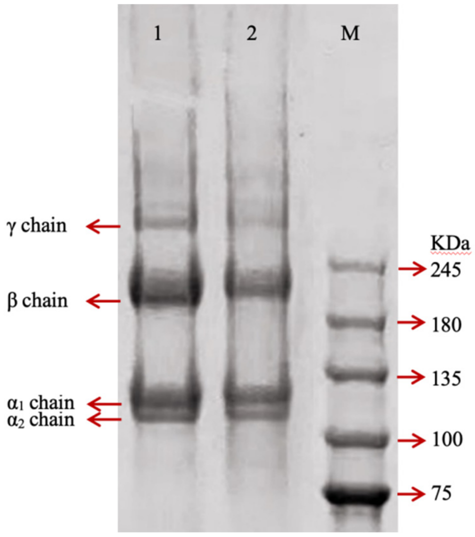

Figure S1 SDS-PAGE of collagen

Note: The three lanes are Marker（M）, collagen + DTT（1）and collagen - DTT（2）
